# 2-Methyl-1-(4-methyl­phenyl­sulfinyl)naphtho­[2,1-*b*]furan

**DOI:** 10.1107/S1600536812003121

**Published:** 2012-01-31

**Authors:** Hong Dae Choi, Pil Ja Seo, Uk Lee

**Affiliations:** a Department of Chemistry, Dongeui University, San 24 Kaya-dong Busanjin-gu, Busan 614-714, Republic of Korea; b Department of Chemistry, Pukyong National University, 599-1 Daeyeon 3-dong, Nam-gu, Busan 608-737, Republic of Korea

## Abstract

In the title compound, C_20_H_16_O_2_S, the 4-methyl­phenyl ring makes a dihedral angle of 82.60 (4)° with the mean plane [r.m.s. deviation = 0.007 (1) Å] of the naphtho­furan fragment. In the crystal, mol­ecules are linked by weak inter­molecular C—H⋯O hydrogen bonds, and by a slipped π–π inter­action between the central naphtho­furan benzene rings of neighbouring mol­ecules [centroid-to-centroid distance = 3.671 (2) Å, inter­planar distance = 3.349 (2) Å and slippage = 1.503 (2)°].

## Related literature

For the pharmacological activity of naphtho­furan compounds, see: Goel & Dixit (2004 ▶); Hagiwara *et al.* (1999 ▶); Piloto *et al.* (2005 ▶). For the crystal structures of related compounds, see: Choi *et al.* (2007 ▶, 2008 ▶).
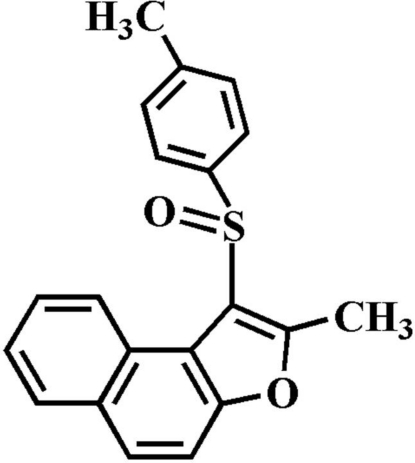


## Experimental

### 

#### Crystal data

C_20_H_16_O_2_S
*M* *_r_* = 320.39
Monoclinic, 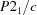

*a* = 6.5052 (1) Å
*b* = 16.7418 (3) Å
*c* = 14.4935 (2) Å
β = 97.883 (1)°
*V* = 1563.55 (4) Å^3^
*Z* = 4
Mo *K*α radiation
μ = 0.21 mm^−1^
*T* = 173 K
0.28 × 0.28 × 0.26 mm

#### Data collection

Bruker SMART APEXII CCD diffractometer
Absorption correction: multi-scan (*SADABS*; Bruker, 2009 ▶) *T*_min_ = 0.677, *T*_max_ = 0.746
15390 measured reflections
3896 independent reflections
3271 reflections with *I* > 2σ(*I*)
*R*_int_ = 0.028

#### Refinement

*R*[*F* ^2^ > 2σ(*F* ^2^)] = 0.039
*wR*(*F* ^2^) = 0.107
*S* = 1.03
3896 reflections
210 parameters
H-atom parameters constrained
Δρ_max_ = 0.29 e Å^−3^
Δρ_min_ = −0.39 e Å^−3^

### 

Data collection: *APEX2* (Bruker, 2009 ▶); cell refinement: *SAINT* (Bruker, 2009 ▶); data reduction: *SAINT*; program(s) used to solve structure: *SHELXS97* (Sheldrick, 2008 ▶); program(s) used to refine structure: *SHELXL97* (Sheldrick, 2008 ▶); molecular graphics: *ORTEP-3* (Farrugia, 1997 ▶) and *DIAMOND* (Brandenburg, 1998 ▶); software used to prepare material for publication: *SHELXL97*.

## Supplementary Material

Crystal structure: contains datablock(s) global, I. DOI: 10.1107/S1600536812003121/lr2049sup1.cif

Structure factors: contains datablock(s) I. DOI: 10.1107/S1600536812003121/lr2049Isup2.hkl

Supplementary material file. DOI: 10.1107/S1600536812003121/lr2049Isup3.cml

Additional supplementary materials:  crystallographic information; 3D view; checkCIF report

## Figures and Tables

**Table 1:** Hydrogen-bond geometry (Å, °)

*D*—H⋯*A*	*D*—H	H⋯*A*	*D*⋯*A*	*D*—H⋯*A*
C6—H6⋯O2^i^	0.95	2.48	3.3211 (19)	147
C10—H10⋯O1^ii^	0.95	2.59	3.4579 (19)	152
C13—H13*C*⋯O2^iii^	0.98	2.35	3.2538 (19)	153

Symmetry codes: (i) 
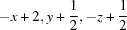
; (ii) 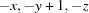; (iii) 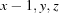.

**Fig. 1.**
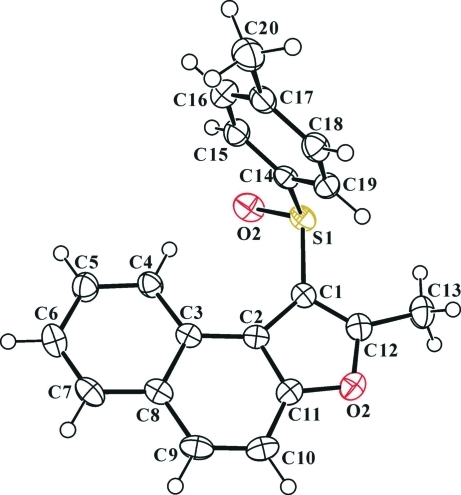
The molecular structure of the title compound. Displacement ellipsoids are drawn at the 50% probability level.

**Fig. 2.**
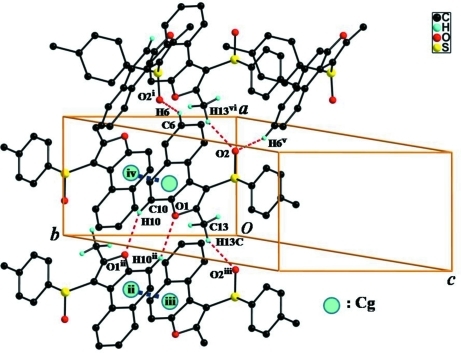
A view of the C—H···O and π–π interactions (dotted lines) in the crystal structure of the title compound. H atoms non-participating in hydrogen-bonding were omitted for clarity. [Symmetry codes: (i) -x + 2, y + 1/2, -z + 1/2; (ii) -x, -y + 1, -z; (iii) x - 1, y, z; (iv) -x + 1, -y + 1, -z; (v) -x + 2, y - 1/2, -z + 1/2; (vi) x + 1, y, z.]

## supplementary crystallographic information

### Comment

Naphthofuran analogues have drawn much attention owing to their valuable biological activities (Goel & Dixit, 2004; Hagiwara *et al.*, 1999; Piloto *et al.*, 2005). As a part of our continuing study of 2-methylnaphthofuran derivatives containing either 1-phenylsulfinyl (Choi *et al.*, 2007) or 1-phenylsulfonyl (Choi *et al.*, 2008) substituents, we report herein the crystal structure of the title compound.

In the title molecule (Fig. 1), the naphthofuran unit is essentially planar, with a mean deviation of 0.007 (1) Å from the least-squares plane defined by the thirteen constituent atoms. The dihedral angle between the 4-methylphenyl ring and the mean plane of the naphthofuran fragment is 82.60 (4)°. The crystal packing (Fig. 2) is stabilized by weak intermolecular C—H···O hydrogen bonds (Table 1). The crystal packing (Fig. 2) is further stabilized by a weak slipped π–π interaction between adjacent central benzene rings of the naphthofuran moiety, with a Cg···Cg^iv^ distance of 3.671 (2) Å, and an interplanar distance of 3.349 (2) Å, resulting in a slippage of 1.503 (2) Å, Cg is the centroid of the C2,C3,C8,C9,C10,C11 benzene ring and (iv) is the -*x* + 1, -*y* + 1, -*z* symmetry code.

### Experimental

77% 3-Chloroperoxybenzoic acid (269 mg, 1.2 mmol) was added in small portions to a stirred solution of 2-methyl-1-(4-methylphenylsulfanyl) naphtho[2,1-b]furan (334 mg, 1.1 mmol) in dichloromethane (30 ml) at 273 K. After being stirred at room temperature for 5 h, the mixture was washed with saturated sodium bicarbonate solution and the organic layer was separated, dried over magnesium sulfate, filtered and concentrated at reduced pressure. The residue was purified by column chromatography (hexane–ethyl acetate, 2:1 v/v) to afford the title compound as a colorless solid [yield 71%, m.p. 433–448 K; *R*_f_ = 0.51 (hexane–ethyl acetate, 2:1 v/v)]. Single crystals suitable for X-ray diffraction were prepared by slow evaporation of a solution of the title compound in ethyl acetate at room temperature.

### Refinement

All H atoms were positioned geometrically and refined using a riding model, with C—H = 0.95 Å for aryl and 0.98 Å for methyl H atoms. *U*_iso_(H) = 1.2*U*_eq_(C) for aryl and 1.5*U*_eq_(C)for methyl H atoms.

### Crystal data

**Table:**

C_20_H_16_O_2_S	*F*(000) = 672
*M**_r_* = 320.39	*D*_x_ = 1.361 Mg m^−^^3^
Monoclinic, *P*2_1_/*c*	Mo *K*α radiation, λ = 0.71073 Å
Hall symbol: -P 2ybc	Cell parameters from 5690 reflections
*a* = 6.5052 (1) Å	θ = 2.8–28.1°
*b* = 16.7418 (3) Å	µ = 0.21 mm^−^^1^
*c* = 14.4935 (2) Å	*T* = 173 K
β = 97.883 (1)°	Block, colourless
*V* = 1563.55 (4) Å^3^	0.28 × 0.28 × 0.26 mm
*Z* = 4	

### Data collection

**Table:**

Bruker SMART APEXII CCD diffractometer	3896 independent reflections
Radiation source: rotating anode	3271 reflections with *I* > 2σ(*I*)
graphite multilayer	*R*_int_ = 0.028
Detector resolution: 10.0 pixels mm^-1^	θ_max_ = 28.3°, θ_min_ = 1.9°
φ and ω scans	*h* = −8→8
Absorption correction: multi-scan (*SADABS*; Bruker, 2009)	*k* = −19→22
*T*_min_ = 0.677, *T*_max_ = 0.746	*l* = −19→19
15390 measured reflections	

### Refinement

**Table:**

Refinement on *F*^2^	Primary atom site location: structure-invariant direct methods
Least-squares matrix: full	Secondary atom site location: difference Fourier map
*R*[*F*^2^ > 2σ(*F*^2^)] = 0.039	Hydrogen site location: difference Fourier map
*wR*(*F*^2^) = 0.107	H-atom parameters constrained
*S* = 1.03	*w* = 1/[σ^2^(*F*_o_^2^) + (0.0548*P*)^2^ + 0.534*P*] where *P* = (*F*_o_^2^ + 2*F*_c_^2^)/3
3896 reflections	(Δ/σ)_max_ < 0.001
210 parameters	Δρ_max_ = 0.29 e Å^−^^3^
0 restraints	Δρ_min_ = −0.39 e Å^−^^3^

### Special details

**Table:**

Geometry. Cg···Cg^iv^ distance = 3.671 (2) Å; (iv) -*x* + 1, -*y* + 1, -*z*Cg is the centroid of the C2/C3/C8–C11 benzene ring.
Refinement. Refinement of F^2^ against ALL reflections. The weighted R-factor wR and goodness of fit S are based on F^2^, conventional R-factors R are based on F, with F set to zero for negative F^2^. The threshold expression of F^2^ > 2sigma(F^2^) is used only for calculating R-factors(gt) etc. and is not relevant to the choice of reflections for refinement. R-factors based on F^2^ are statistically about twice as large as those based on F, and R- factors based on ALL data will be even larger.

### Fractional atomic coordinates and isotropic or equivalent isotropic displacement parameters (Å^2^)

**Table:**

	*x*	*y*	*z*	*U*_iso_*/*U*_eq_	
S1	0.54869 (6)	0.29240 (2)	0.22725 (3)	0.02964 (11)	
O1	0.18369 (15)	0.42901 (7)	0.06023 (7)	0.0332 (2)	
O2	0.77697 (17)	0.28641 (6)	0.22477 (8)	0.0391 (3)	
C1	0.4389 (2)	0.37111 (8)	0.15685 (9)	0.0268 (3)	
C2	0.4942 (2)	0.45457 (8)	0.14760 (9)	0.0251 (3)	
C3	0.6672 (2)	0.50427 (8)	0.17904 (9)	0.0253 (3)	
C4	0.8502 (2)	0.47737 (9)	0.23408 (10)	0.0301 (3)	
H4	0.8619	0.4230	0.2528	0.036*	
C5	1.0110 (2)	0.52903 (9)	0.26072 (12)	0.0368 (3)	
H5	1.1337	0.5100	0.2972	0.044*	
C6	0.9958 (2)	0.60987 (9)	0.23450 (12)	0.0375 (4)	
H6	1.1067	0.6455	0.2544	0.045*	
C7	0.8224 (2)	0.63704 (9)	0.18063 (11)	0.0339 (3)	
H7	0.8146	0.6917	0.1629	0.041*	
C8	0.6534 (2)	0.58600 (8)	0.15034 (10)	0.0282 (3)	
C9	0.4756 (2)	0.61434 (9)	0.09111 (10)	0.0338 (3)	
H9	0.4694	0.6691	0.0736	0.041*	
C10	0.3145 (2)	0.56566 (10)	0.05886 (10)	0.0346 (3)	
H10	0.1974	0.5847	0.0185	0.042*	
C11	0.3302 (2)	0.48621 (9)	0.08821 (10)	0.0289 (3)	
C12	0.2541 (2)	0.35928 (9)	0.10221 (10)	0.0309 (3)	
C13	0.1180 (3)	0.28882 (10)	0.08024 (12)	0.0399 (4)	
H13A	0.1879	0.2409	0.1079	0.060*	
H13B	0.0887	0.2822	0.0125	0.060*	
H13C	−0.0124	0.2967	0.1058	0.060*	
C14	0.5188 (2)	0.33407 (8)	0.33886 (9)	0.0254 (3)	
C15	0.6874 (2)	0.33510 (9)	0.40837 (11)	0.0330 (3)	
H15	0.8197	0.3177	0.3958	0.040*	
C16	0.6611 (2)	0.36179 (10)	0.49654 (11)	0.0367 (3)	
H16	0.7769	0.3626	0.5442	0.044*	
C17	0.4692 (2)	0.38742 (9)	0.51671 (10)	0.0318 (3)	
C18	0.3013 (2)	0.38436 (9)	0.44579 (10)	0.0316 (3)	
H18	0.1685	0.4011	0.4585	0.038*	
C19	0.3236 (2)	0.35751 (8)	0.35733 (10)	0.0289 (3)	
H19	0.2072	0.3551	0.3100	0.035*	
C20	0.4422 (3)	0.41837 (11)	0.61182 (11)	0.0416 (4)	
H20A	0.5394	0.3909	0.6590	0.062*	
H20B	0.2997	0.4084	0.6236	0.062*	
H20C	0.4700	0.4759	0.6147	0.062*	

### Atomic displacement parameters (Å^2^)

**Table:**

	*U*^11^	*U*^22^	*U*^33^	*U*^12^	*U*^13^	*U*^23^
S1	0.0360 (2)	0.02007 (18)	0.0343 (2)	0.00179 (13)	0.00981 (14)	−0.00098 (13)
O1	0.0290 (5)	0.0398 (6)	0.0299 (5)	0.0007 (4)	0.0012 (4)	−0.0028 (4)
O2	0.0378 (6)	0.0329 (6)	0.0494 (7)	0.0121 (5)	0.0161 (5)	0.0032 (5)
C1	0.0293 (7)	0.0260 (7)	0.0259 (7)	−0.0002 (5)	0.0068 (5)	−0.0021 (5)
C2	0.0282 (6)	0.0246 (7)	0.0232 (6)	0.0036 (5)	0.0066 (5)	−0.0009 (5)
C3	0.0295 (7)	0.0231 (7)	0.0245 (6)	0.0023 (5)	0.0079 (5)	−0.0015 (5)
C4	0.0299 (7)	0.0235 (7)	0.0372 (8)	0.0009 (5)	0.0058 (6)	0.0014 (6)
C5	0.0304 (7)	0.0335 (8)	0.0458 (9)	−0.0007 (6)	0.0027 (6)	0.0006 (7)
C6	0.0354 (8)	0.0310 (8)	0.0475 (9)	−0.0082 (6)	0.0110 (7)	−0.0053 (7)
C7	0.0440 (8)	0.0223 (7)	0.0384 (8)	−0.0018 (6)	0.0158 (7)	−0.0008 (6)
C8	0.0362 (7)	0.0237 (7)	0.0265 (7)	0.0023 (5)	0.0114 (6)	−0.0009 (5)
C9	0.0449 (8)	0.0266 (7)	0.0312 (7)	0.0101 (6)	0.0101 (6)	0.0046 (6)
C10	0.0372 (8)	0.0365 (8)	0.0295 (7)	0.0117 (6)	0.0023 (6)	0.0039 (6)
C11	0.0285 (7)	0.0326 (7)	0.0259 (7)	0.0027 (6)	0.0049 (5)	−0.0023 (6)
C12	0.0322 (7)	0.0336 (8)	0.0281 (7)	−0.0011 (6)	0.0086 (6)	−0.0044 (6)
C13	0.0371 (8)	0.0427 (9)	0.0402 (9)	−0.0106 (7)	0.0061 (7)	−0.0088 (7)
C14	0.0293 (7)	0.0188 (6)	0.0285 (7)	0.0001 (5)	0.0054 (5)	0.0029 (5)
C15	0.0266 (7)	0.0340 (8)	0.0383 (8)	0.0012 (6)	0.0041 (6)	0.0032 (6)
C16	0.0325 (7)	0.0423 (9)	0.0338 (8)	−0.0025 (6)	−0.0016 (6)	0.0031 (7)
C17	0.0374 (8)	0.0288 (7)	0.0299 (7)	−0.0075 (6)	0.0070 (6)	0.0017 (6)
C18	0.0291 (7)	0.0315 (8)	0.0354 (8)	0.0003 (6)	0.0090 (6)	0.0010 (6)
C19	0.0267 (7)	0.0285 (7)	0.0311 (7)	0.0007 (5)	0.0029 (5)	0.0020 (6)
C20	0.0502 (9)	0.0444 (9)	0.0318 (8)	−0.0107 (8)	0.0111 (7)	−0.0035 (7)

### Geometric parameters (Å, °)

**Table:**

S1—O2	1.4938 (11)	C9—H9	0.9500
S1—C1	1.7573 (15)	C10—C11	1.396 (2)
S1—C14	1.7964 (14)	C10—H10	0.9500
O1—C12	1.3662 (18)	C12—C13	1.483 (2)
O1—C11	1.3727 (17)	C13—H13A	0.9800
C1—C12	1.360 (2)	C13—H13B	0.9800
C1—C2	1.4537 (19)	C13—H13C	0.9800
C2—C11	1.3813 (19)	C14—C15	1.384 (2)
C2—C3	1.4232 (19)	C14—C19	1.3901 (18)
C3—C4	1.413 (2)	C15—C16	1.386 (2)
C3—C8	1.4293 (19)	C15—H15	0.9500
C4—C5	1.371 (2)	C16—C17	1.389 (2)
C4—H4	0.9500	C16—H16	0.9500
C5—C6	1.406 (2)	C17—C18	1.394 (2)
C5—H5	0.9500	C17—C20	1.505 (2)
C6—C7	1.359 (2)	C18—C19	1.385 (2)
C6—H6	0.9500	C18—H18	0.9500
C7—C8	1.414 (2)	C19—H19	0.9500
C7—H7	0.9500	C20—H20A	0.9800
C8—C9	1.424 (2)	C20—H20B	0.9800
C9—C10	1.359 (2)	C20—H20C	0.9800
			
O2—S1—C1	111.27 (6)	O1—C11—C10	123.78 (13)
O2—S1—C14	106.16 (7)	C2—C11—C10	124.97 (14)
C1—S1—C14	98.40 (6)	C1—C12—O1	110.52 (13)
C12—O1—C11	106.72 (11)	C1—C12—C13	134.00 (15)
C12—C1—C2	107.26 (13)	O1—C12—C13	115.48 (13)
C12—C1—S1	119.03 (11)	C12—C13—H13A	109.5
C2—C1—S1	133.49 (11)	C12—C13—H13B	109.5
C11—C2—C3	118.98 (13)	H13A—C13—H13B	109.5
C11—C2—C1	104.24 (12)	C12—C13—H13C	109.5
C3—C2—C1	136.67 (13)	H13A—C13—H13C	109.5
C4—C3—C2	124.22 (13)	H13B—C13—H13C	109.5
C4—C3—C8	118.95 (13)	C15—C14—C19	120.66 (13)
C2—C3—C8	116.82 (13)	C15—C14—S1	119.20 (10)
C5—C4—C3	120.60 (14)	C19—C14—S1	119.84 (11)
C5—C4—H4	119.7	C14—C15—C16	119.32 (13)
C3—C4—H4	119.7	C14—C15—H15	120.3
C4—C5—C6	120.57 (15)	C16—C15—H15	120.3
C4—C5—H5	119.7	C15—C16—C17	121.45 (14)
C6—C5—H5	119.7	C15—C16—H16	119.3
C7—C6—C5	119.97 (14)	C17—C16—H16	119.3
C7—C6—H6	120.0	C16—C17—C18	117.99 (14)
C5—C6—H6	120.0	C16—C17—C20	121.35 (14)
C6—C7—C8	121.65 (14)	C18—C17—C20	120.66 (14)
C6—C7—H7	119.2	C19—C18—C17	121.58 (13)
C8—C7—H7	119.2	C19—C18—H18	119.2
C7—C8—C9	121.24 (13)	C17—C18—H18	119.2
C7—C8—C3	118.23 (13)	C18—C19—C14	118.97 (13)
C9—C8—C3	120.51 (13)	C18—C19—H19	120.5
C10—C9—C8	122.13 (14)	C14—C19—H19	120.5
C10—C9—H9	118.9	C17—C20—H20A	109.5
C8—C9—H9	118.9	C17—C20—H20B	109.5
C9—C10—C11	116.49 (14)	H20A—C20—H20B	109.5
C9—C10—H10	121.8	C17—C20—H20C	109.5
C11—C10—H10	121.8	H20A—C20—H20C	109.5
O1—C11—C2	111.24 (12)	H20B—C20—H20C	109.5
			
O2—S1—C1—C12	137.65 (11)	C12—O1—C11—C10	179.24 (13)
C14—S1—C1—C12	−111.29 (12)	C3—C2—C11—O1	176.11 (11)
O2—S1—C1—C2	−48.61 (15)	C1—C2—C11—O1	−0.85 (14)
C14—S1—C1—C2	62.45 (14)	C3—C2—C11—C10	−3.1 (2)
C12—C1—C2—C11	1.38 (14)	C1—C2—C11—C10	179.93 (13)
S1—C1—C2—C11	−172.89 (11)	C9—C10—C11—O1	−178.33 (13)
C12—C1—C2—C3	−174.74 (15)	C9—C10—C11—C2	0.8 (2)
S1—C1—C2—C3	11.0 (2)	C2—C1—C12—O1	−1.46 (15)
C11—C2—C3—C4	−175.09 (12)	S1—C1—C12—O1	173.79 (9)
C1—C2—C3—C4	0.6 (2)	C2—C1—C12—C13	179.00 (15)
C11—C2—C3—C8	3.32 (18)	S1—C1—C12—C13	−5.8 (2)
C1—C2—C3—C8	179.02 (14)	C11—O1—C12—C1	0.93 (15)
C2—C3—C4—C5	179.31 (13)	C11—O1—C12—C13	−179.43 (12)
C8—C3—C4—C5	0.9 (2)	O2—S1—C14—C15	−15.36 (13)
C3—C4—C5—C6	0.6 (2)	C1—S1—C14—C15	−130.48 (12)
C4—C5—C6—C7	−1.4 (2)	O2—S1—C14—C19	170.95 (11)
C5—C6—C7—C8	0.6 (2)	C1—S1—C14—C19	55.83 (12)
C6—C7—C8—C9	−177.67 (14)	C19—C14—C15—C16	−1.7 (2)
C6—C7—C8—C3	1.0 (2)	S1—C14—C15—C16	−175.29 (12)
C4—C3—C8—C7	−1.69 (19)	C14—C15—C16—C17	0.1 (2)
C2—C3—C8—C7	179.81 (12)	C15—C16—C17—C18	1.0 (2)
C4—C3—C8—C9	176.95 (12)	C15—C16—C17—C20	−178.40 (15)
C2—C3—C8—C9	−1.56 (18)	C16—C17—C18—C19	−0.7 (2)
C7—C8—C9—C10	177.87 (14)	C20—C17—C18—C19	178.78 (14)
C3—C8—C9—C10	−0.7 (2)	C17—C18—C19—C14	−0.8 (2)
C8—C9—C10—C11	1.2 (2)	C15—C14—C19—C18	2.0 (2)
C12—O1—C11—C2	0.01 (15)	S1—C14—C19—C18	175.61 (11)

### Hydrogen-bond geometry (Å, °)

**Table:**

*D*—H···*A*	*D*—H	H···*A*	*D*···*A*	*D*—H···*A*
C6—H6···O2^i^	0.95	2.48	3.3211 (19)	147.
C10—H10···O1^ii^	0.95	2.59	3.4579 (19)	152.
C13—H13C···O2^iii^	0.98	2.35	3.2538 (19)	153.

Symmetry codes: (i) −*x*+2, *y*+1/2, −*z*+1/2; (ii) −*x*, −*y*+1, −*z*; (iii) *x*−1, *y*, *z*.

## References

[bb1] Brandenburg, K. (1998). *DIAMOND* Crystal Impact GbR, Bonn, Germany.

[bb2] Bruker (2009). *APEX2*, *SADABS* and *SAINT* Bruker AXS Inc., Madison, Wisconsin, USA.

[bb3] Choi, H. D., Seo, P. J., Son, B. W. & Lee, U. (2007). *Acta Cryst.* E**63**, o1731–o1732.

[bb4] Choi, H. D., Seo, P. J., Son, B. W. & Lee, U. (2008). *Acta Cryst.* E**64**, o727. 10.1107/S1600536808007046 PMC2961025 21202117

[bb5] Farrugia, L. J. (1997). *J. Appl. Cryst.* **30**, 565.

[bb6] Goel, A. & Dixit, M. (2004). *Tetrahedron Lett* **45**, 8819–8821.

[bb7] Hagiwara, H., Sato, K., Suzuki, T. & Ando, M. (1999). *Heterocycles*, **51**, 497–500.

[bb8] Piloto, A. M., Costa, S. P. G. & Goncalves, M. S. T. (2005). *Tetrahedron Lett.* **46**, 4757–4760.

[bb9] Sheldrick, G. M. (2008). *Acta Cryst.* A**64**, 112–122. 10.1107/S0108767307043930 18156677

